# Guanabenz Prevents d-Galactosamine/Lipopolysaccharide-Induced Liver Damage and Mortality

**DOI:** 10.3389/fimmu.2017.00679

**Published:** 2017-06-13

**Authors:** Jessica Perego, Clarisse Bourbon, Lionel Chasson, Caroline Laprie, Lionel Spinelli, Voahirana Camosseto, Evelina Gatti, Philippe Pierre

**Affiliations:** ^1^CIML, Aix-Marseille University, CNRS, INSERM, Marseille, France; ^2^Aveiro Health Sciences Program, Institute for Research in Biomedicine (iBiMED), University of Aveiro, Aveiro, Portugal; ^3^International Associated Laboratory (LIA), CNRS “Mistra”, Marseille, France

**Keywords:** guanabenz, lipopolysaccharide-induced liver damage, TLR4, unfolded protein response, Ppp1r15a

## Abstract

Multi-organ failure in response to uncontrolled microbial infection is characterized by low blood pressure accompanied by a systemic over-inflammation state, caused by massive pro-inflammatory cytokines release and liver damage. Recently, the integrated stress response (ISR), characterized by eukaryotic translation initiation factor 2α (eIF2α) phosphorylation, was involved with controlling apoptosis in stressed hepatocytes and associated with poor survival to endotoxin challenge. Lipopolysaccharide (LPS) alone is able to induce the ISR in hepatocytes and can trigger massive liver damage along with tumor necrosis factor-alpha (TNF-α) expression. Consequently, drugs interfering with eIF2α phosphorylation may represent potential candidates for the treatment of such pathologies. We, therefore, used Guanabenz (GBZ), a small compound with enhancing eIF2α phosphorylation activity to evaluate its effect on bacterial LPS sensing and endotoxemia. GBZ is confirmed here to have an anti-inflammatory activity by increasing *in vitro* interleukin-10 (IL-10) production by LPS-stimulated dendritic cells. We further show that in the d-galactosamine (d-galN)/LPS-dependent lethality model, intraperitoneal injection of GBZ promoted mice survival, prevented liver damage, increased IL-10 levels, and inhibited TNF-α production. GBZ and its derivatives could therefore represent an interesting pharmacological solution to control systemic inflammation and associated acute liver failure.

## Introduction

Endotoxemia is a complex syndrome defined by an uncontrolled activation of the innate immune system, with initiation of the complement pathway and high amounts of circulating pro-inflammatory tumor necrosis factor-alpha (TNF-α), which induces multiple organs failure (1). Toll-like receptors (TLRs) play therefore an important role in sepsis through pathogen recognition and TNF-α induction (1). TLR4, which recognizes bacterial lipopolysaccharide (LPS), is the most implicated TLR in septic syndrome progression, *via* activation of both myeloid differentiation primary-response protein 88 (MyD88)-dependent signaling pathways (2). Systemic TLR4 triggering is often the result of microbe translocation from the gut lumen into the blood stream, which is normally reduced by a collaboration between the intestinal tract cells and associated immunocytes. In this situation, the liver constitutes the second line of defense by eliminating the remaining invading bacteria and bacterial products (e.g., LPS), and preventing systemic dispersion. Liver injury before or after the onset of sepsis has therefore a critical effect on the severity and outcome of the disease (3). Accordingly, patients with liver cirrhosis are at greater risk of succumbing to bacterial infections, since liver dysfunction amplifies systemic and lung inflammatory responses, causing respiratory failures (4). Liver dysfunction often occurs in early sepsis, and one of the classical experimental mouse model of endotoxemia is based on liver sensitization by d-galactosamine (d-galN) prior to LPS injection (5). In this model, however, LPS-induced lethality has been shown to be triggered by a caspase-dependent fulminant apoptotic hepatitis induced by TNF-α overproduction and not directly from the systemic inflammatory response (6).

Recently, studies performed in mouse and septic patients’ tissues underlined the role of the integrated stress response (ISR), as one of the main causes of apoptosis in hepatocytes during systemic inflammation (7). ISR-induced cell death involves sequential steps of PKR-like endoplasmic reticulum kinase (PERK)-mediated eukaryotic initiation factor α (eIF2α) phosphorylation. eIF2α phosphorylation causes a transient but profound reduction of protein synthesis, and the expression of activating transcription factor 4 (ATF4) and of the proapoptotic CCAAT/enhancer binding protein (C/EBP) homologous protein (CHOP) (8). ATF4 synthesis and eIF2α phosphorylation, in turn, promote growth arrest and DNA damage-inducible protein 34 (GADD34/Ppp1r15a) expression, a regulatory subunit of the protein phosphatase 1 (PP1c). GADD34 association with PP1c acts as a negative feedback regulator that terminates eIF2α phosphorylation and restore translation. Interestingly, GADD34 was shown to have both a positive and a negative role in liver regeneration after stress-induced damage (9, 10). Independent of its negative feedback role in the ISR, we recently reported that eIF2α dephosphorylation is necessary for the normal pro-inflammatory cytokine production observed after TLRs stimulation by different microbial agonists (11–13). Thus, interfering with the eIF2α phosphorylation/dephosphorylation cycle could represent an interesting pharmacological strategy to limit systemic inflammation and liver failure during endotoxemia.

Guanabenz [GBZ, 2-(2,6-dichlorobenzylidene)-hydrazinecarboximidamide] has been recently shown to display specific eIF2α dephosphorylation inhibitory activity (14) and was used to protect cells from ISR-dependent apoptosis in different clinical models (15–17). Originally, GBZ was developed, as a well-tolerated small α2-adrenergic receptor agonist that received FDA approval (Wytensin) for the treatment of hypertension (18), and represents therefore an interesting chemical compound to evaluate the therapeutic interest of targeting the eIF2α phosphorylation pathway, with the aim of controlling inflammation and prevent organs failure. Given the lethal consequences of TNF-α-mediated inflammation and liver apoptosis after injection of low doses of LPS in d-galN sensitized mice, this model appeared to be particularly well suited to test GBZ capacity to prevent acute liver failure and LPS-induced inflammatory response. Here, using DNA microarray analysis and immunoassays, we show that GBZ treatment alters LPS-dependent cytokine production both *in vitro* and *in vivo* and triggers a spectacular rescue of mice viability in the d-galN/LPS model. GBZ seems to exert this effect both through a strong protection against TNF-α-mediated liver apoptosis and by skewing the cytokine response toward anti-inflammatory interleukin-10 (IL-10) production.

## Materials and Methods

### Mice and Ethics Statement

This study was carried out in strict accordance with the recommendations in the Guide for the Care and Use of Laboratory Animals the French Ministry of Agriculture and of the European Union. Mice were housed under specific pathogen-free conditions and handled in accordance with French and European directives. Animals were housed in the CIML animal facilities accredited by the French Ministry of Agriculture to perform experiments on alive mice. All animal experiments were approved by Direction Départementale des Services Vétérinaires des Bouches du Rhône (Approval number A13-543). All efforts were made to minimize animal suffering. The experiments were performed with C57BL/6 mice purchased from Janvier. Sex and age were varying according to the experiment performed.

### Cell Culture

BM-derived dendritic cells (DCs) were differentiated *in vitro* from the bone marrow of 6–8-week-old male mice, using GM-CSF, produced using J558L cells. Bone marrow progenitors were plated at 0.8×10^6^ cells/ml, 5 ml/well in 6-well plates, and cultivated with RPMI (GIBCO), 5% FCS (Sigma-Aldrich), 20 µg/ml gentamycin (Sigma-Aldrich), 50 µM β-mercaptoethanol (VWR), and GM-CSF. The medium was replaced every 2 days; BM-derived DCs were used for experiments at day 6.

### Reagents

Lipopolysaccharide (*Escherichia coli* O55:B5, Sigma-Aldrich L2880), clonidine hydrochloride, noradrenaline l, and d-(+) galactosamine hydrochloride are from Sigma-Aldrich; GBZ is from Tocris Bioscience; poly I:C high molecular weight is from Invivogen.

### Cytokines Measurement

Mouse TNF-α, IL-6, IL-12, and IL-10 were quantified using ELISA kit (eBioscience), according to manufacturer instructions. For the detection of cytokines in the blood stream, blood sera were collected 1 and 3 h after the injection of LPS, using an 18-gauge needle, from the cheek.

### Immunoblotting

Cells were lysed in 1% Triton X-100, 50 mM Hepes, 10 mM NaCl, 2.5 mM MgCl_2_, 2 mM EDTA, 10% glycerol, supplemented with Complete Mini Protease Inhibitor Cocktail Tablets (Roche). Protein quantification was performed using the BCA Protein Assay (Pierce). 20–25 µg of Triton X-100-soluble material were loaded on 10% SDS-PAGE before immunoblotting and chemiluminescence detection (SuperSignal West Pico Chemiluminescent Substrate, Pierce). Rabbit polyclonal antibodies against eIF2α (D7D3) was purchased from Cell Signaling Technologies. Rabbit polyclonal antibody against p-eIF2α (Ser51) was from Abcam. Secondary antibodies were purchased from Jackson ImmunoResearch Laboratories.

### Immunohistochemistry and Pathology Analysis

Mouse liver was collected 48 h after LPS injection or immediately after the animal’s death. The organs were fixed in 10% buffered formalin for 24 h, and then, the tissues were dehydrated and embedded in paraffin. Tissue sections of 3.5 µm were cut using the microtom Leica RM2245. Hematoxylin-eosin staining was effectuated automatically with Leica autostainer XL. Finally, the slides were mounted with entellan and kept at room temperature. Biopsies were analyzed by an anatomopathologist and the clinical score was assigned in a blinded way, following the parameters reported in the study by Siegmund et al. (19). Pictures were taken with Nikon Eclipse.

The total score for acute hepatitis is given by the sum of the inflammation score (portal + lobular scores) and the necrosis score. Here are the details for both parameters:

#### *Inflammation Score*:

0—no inflammation
1—mild lobular inflammation (<10% of liver parenchyma)/mild portal inflammation (<1/3 of portal tracts)
2—moderate lobular inflammation (10–50% of liver parenchyma)/moderate portal inflammation (approximately 50% of portal tracks)
3—severe lobular inflammation (>50% of liver parenchyma)/severe portal inflammation (>2/3 of portal tracts).

#### *Necrosis Score*:

0—no necrosis
1—<10% necrosis of liver parenchyma
2—10–25% necrosis of liver parenchyma
3—>25% necrosis of liver parenchyma.

The immunohistochemistry detection of cleaved caspase 3 was carried out on deparaffinized sections. Before staining, sections were subjected to heat-induced epitope retrieval by incubation in a 0.01 M sodium citrate solution (pH 6) at 95°C for 30 min, followed by a 30 min cool down. Primary antibodies were diluted in the following buffer: PBS, 2% (*m/v*) BSA, 0.05% saponin. Active caspase 3 was detected with a rabbit polyclonal antibody purchased from Cell Signaling Technology. The primary antibody was applied for 1 h at room temperature. The sections were then washed and incubated for 1 h at room temperature with the ImmPRESS™ HRP Anti-Rabbit IgG (Peroxidase) Polymer Detection Kit, purchased from Vector Laboratories. Bound peroxidase was identified using 3,3′-diaminobenzidine (DAB Peroxidase HRP Substrate Kit with Nickel, Vector Laboratories). Nuclear counterstaining was performed with hematoxylin. Pictures were taken with Nikon Eclipse, 20× objective. For detecting apoptosis, paraffin-embedded slides were deparaffinized and TUNEL staining was performed using DeadEnd Fluorometric TUNEL System (Promega), following the manufacturer’s instructions. The images were taken with the confocal microscope LSM580 (Carl Zeiss), 20× objective and accompanying imaging software.

### *In Vivo* Experiments

Wild-type female mice C57BL/6 of 8 weeks were injected i.p. with 2 mg/kg of GBZ, clonidine or PBS/DMSO. One hour later, they were injected i.p. with 20 mg/mouse of d-galN and 20 ng/20 g of mouse of LPS (or PBS). When used, noradrenaline was injected i.p. at 0.5 mg/kg. For some experiments, the order of the injections was modified (specified in the text). In our initial experimental conditions (*n* = 15 survey over 5 days), we never observed any additional death after the 48 h time point, with the majority of the deaths occurring within 24 h. Mice survival was, therefore, checked every 12 h for up to 48 h. For some experiments, blood sera were collected 1 and 3 h after the injection of d-galN and serum was used to screen circulating cytokines (TNF-α and IL-10). Similar blood volumes were collected only once from different individual animals that were all injected at the same time, with the same solutions, in the same conditions. Blood-drawn animals were not included in the survival statistics. For some experiments, the liver was collected for immunohistochemistry after 2 days or immediately upon animals’ death. For i.p. injections, 1 ml syringes and 25 gauge needles were used.

### Gene Expression Analysis

Total RNA was isolated with RNeasy kit (Qiagen). cDNA was synthesized with random hexamers and superscript II reverse transcriptase (Invitrogen). For Affymetrix microarray analysis, GM-CSF DCs were cultured in RPMI supplemented with 5% heat-inactivated fetal bovine serum, 50 µM beta-mercaptoethanol and GM-CSF. Cells were differentiated for 6 days before the experiment. Cells were treated for 8 h with *Proteus mirabilis* and harvested before lysis. Control and *P. mirabilis* treated DCs were incubated with the bacteriostatic chloramphenicol to avoid bacterial overgrowth. GBZ was used at 50 µM and added at the same time as *P. mirabilis*. Each condition was analyzed in independent triplicates (*n* = 3). Hybridization to arrays (Affymetrix GeneChip Mouse Gene 1.0 ST) and image scanning were performed according to the Affymetrix Expression Analysis Technical Manual. Gene Expression microarray raw data were normalized using limmaGUI software (R/Bioconductor, Boston, MA, USA). Data can be accessed through the GEO repository accession number GSE90831. Microarrays data were analyzed with the ingenuity pathway analysis (IPA) software (Qiagen). Quantitative real time PCR analysis was performed with Applied Biosystems PRISM 7700 Sequence Detection System. The primers used are: mouse IL-10 forward: 5′-CAGCAGACTCAATACACACT-3′; mouse IL-10 reverse: 5′-TGGCCCAGAAATCAAGGAGC-3′; mouse RPS16 (housekeeping) forward: 5′-CTTGGATTCATCCACATA-3′; mouse RPS16 reverse: 5′-ATTTGCTGGTGTGGATATTCG-3′.

### Statistical Analysis

Statistical analysis for cytokines levels and liver damage were performed with GraphPad Prism software. When several conditions were to compare, we performed a one-way ANOVA, followed by Tukey range test to assess the significance among pairs of conditions. When only two conditions were to test, we performed Student’s *t*-test or Welch *t*-test (according to the validity of homoscedasticity hypothesis). Statistical analysis on survival curves were performed using log-rank test, followed by Benjamini–Yekutieli correction when required.

## Results

### GBZ Impacts LPS-Dependent Cytokine Expression in DCs

**Figure 1:**
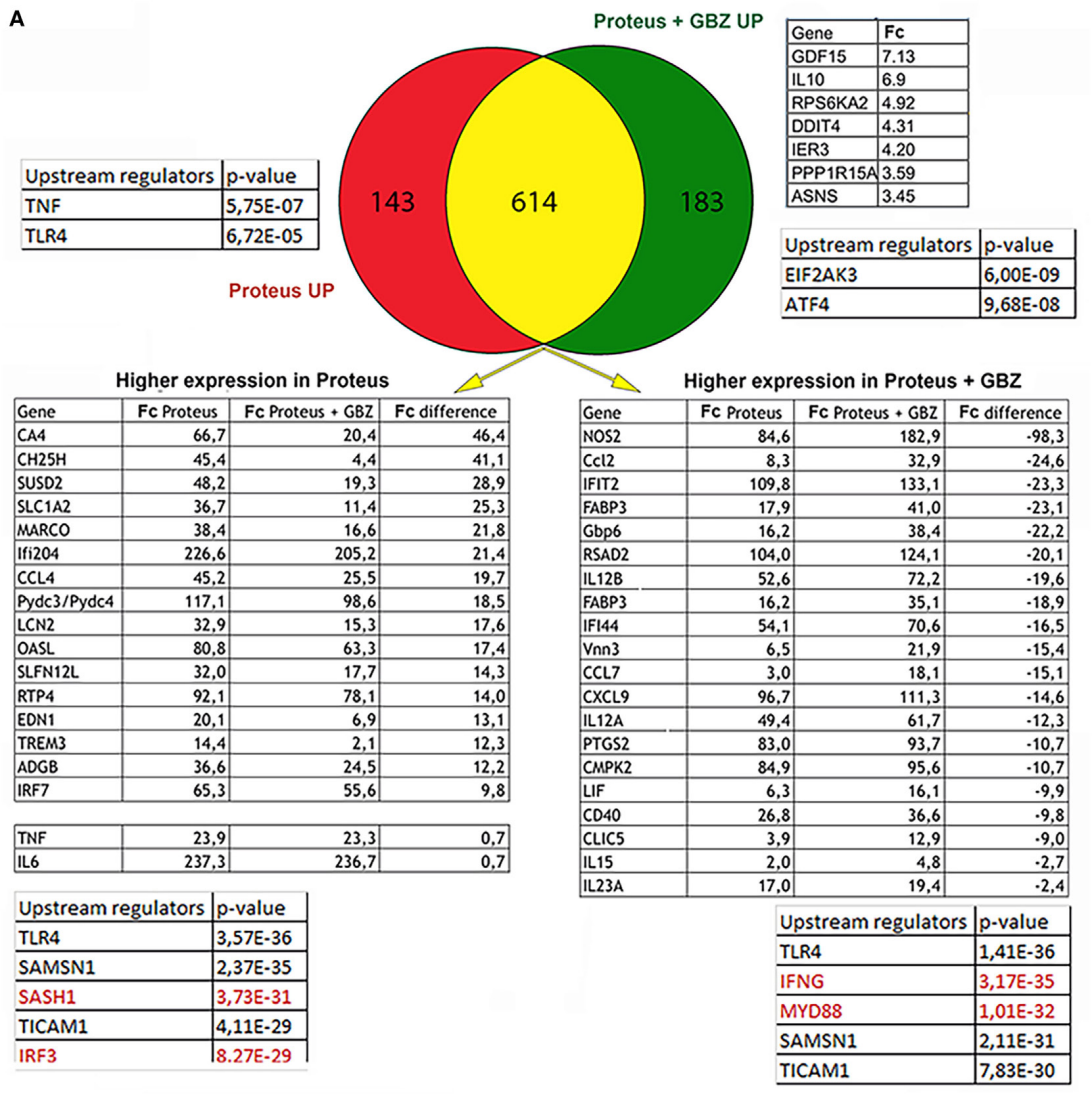

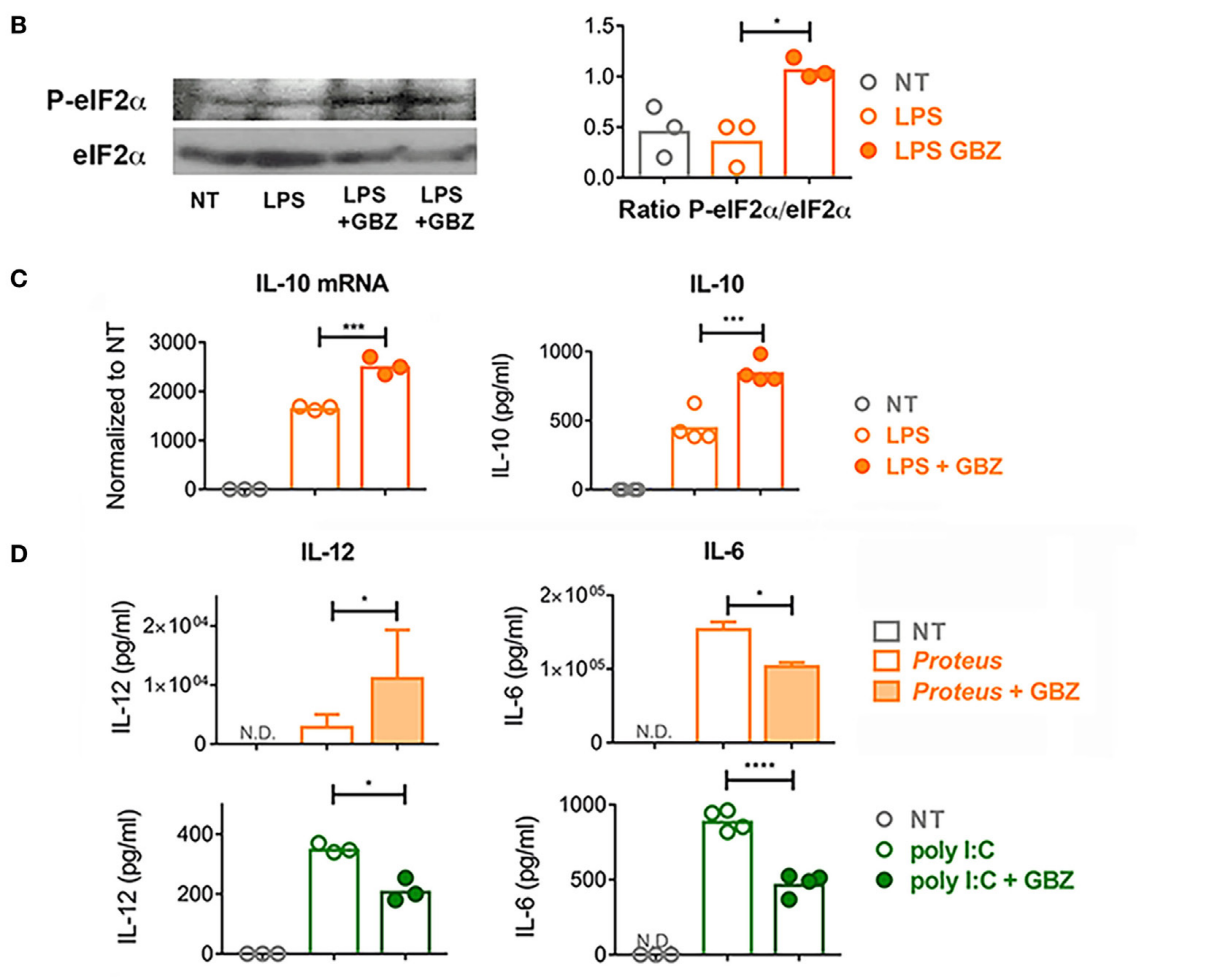
Guanabenz (GBZ) immunomodulatory effect on TLR4 signaling. **(A)** GM-CSF-derived dendritic cells (DCs) were stimulated for 8 h with *Proteus mirabilis* (0.1 MOI), in the presence or absence of GBZ (50 µM). Gene expression was established by Affymetrix microarray analysis and compared with the ingenuity pathway analysis (IPA) Ingenuity Pathway software. Venn diagram was used to separate DEGs in three subsets. Tables contain selected DEGs for each subset and displaying the highest difference in expression level compared to control groups. Major upstream regulators identified by IPA are presented for each subset or group. **(B)** GM-CSF-derived DCs were treated for 6 h with 100 ng/ml lipopolysaccharide (LPS) 055:B5, in the presence or absence of GBZ (50 µM). Protein lysates were blotted for P-eIF2α and total eIF2α. The ratio, calculated in three independent experiments, was plotted. **(C)** On the same samples, the level of interleukin-10 (IL-10) transcription and secretion was measured by qPCR (*n* = 3) and ELISA (*n* = 4) **(D)**. GM-CSF-derived DCs were treated for 8 h with *P. mirabilis* (0.1 MOI) or 10 µg/ml of poly I:C, with or without GBZ (50 µM). The secretion of IL-12 (*n* = 3) and IL-6 (*n* = 4) was measured by ELISA. Statistical significance was assigned using one-way ANOVA test followed by Tukey range test to assess the significance among pairs of conditions (*p < 0.05; ***p < 0.001; ****p < 0.0001).

It has been demonstrated that components of the ISR have an active role in controlling cytokines expression (20). GBZ by preventing eIF2-α dephosphorylation impacts directly the ISR (14) and has, thus, potentially the capacity of altering cytokines expression in cells exposed to microbial stimuli. Given the role of DCs in bridging innate and adaptive immunity by coordinating antigen presentation with pro-inflammatory cytokines and type-I interferon production, we first tested the consequences of GBZ treatment on bone marrow-derived DCs activated with the gram-negative bacteria *P. mirabilis*. *P. mirabilis* is one of the most common pathogens encountered in clinical specimens and can cause a variety of hospital-acquired illnesses, including urinary tract or bloodstream infections. We performed an unbiased comparative DNA microarray-based analysis on purified CD11c+ DCs, stimulated with chloramphenicol treated-*P. mirabilis* in the presence or absence of GBZ, to have a comprehensive view of the transcription landscape altered by GBZ during the innate response to a common pathogen. Venn diagram analysis (Figure 1A) revealed that among the 183 genes uniquely induced in the presence of GBZ (DEGs > 2 folds), we could not define, using IPA, any statistically relevant group or pathway being linked to inflammation. However, we could single-out the *IL-10* gene transcription, as being strongly enhanced after *P. mirabilis* sensing in the presence of GBZ. Among the top upstream regulators, for this gene group, eIF2AK3 (PERK) and ATF4 were identified, which suggested that GBZ treatment, as expected, was causing an enhancement of eIF2α-phosphorylation, promoting ISR activation. Immunoblot analysis confirmed this situation with P-eIF2α levels being increased by GBZ treatment in LPS-activated DCs (Figure 1B).

As for the 143 genes only upregulated in response to *Proteus*, no clear pathway identification and relatively low statistical score for TNF-α and TLR4 as upstream regulators were given by the IPA. We, therefore, focused on the larger pool of commonly up-regulated genes (614) in which pro-inflammatory cytokines expression was, in most cases, slightly augmented by GBZ. However, expression of several genes was also severely affected, including nitric oxide synthase 2 (*NOS2*) and cholesterol 25 hydroxylase (*Ch25h*) that were either strongly or poorly induced after DC co-exposure to *P. mirabilis* and GBZ. Given these heterogenous variations observed among commonly upregulated genes, we formed two groups of DEGs defined by their augmented or reduced induction in the presence of GBZ, and ran an IPA on these two groups. Among the identified upstream regulators, the most relevant for the two groups was TLR4, which confirmed that the clear majority of DEGs identified in these experiments using intact bacteria, were similar to the one previously found induced in response to purified *E. coli* LPS (21). However, while the DEGs with an expression reinforced by GBZ, belonged to pathways regulated by MyD88 and IFN γ, those with a reduced expression appeared to be controlled by IRF3 and SASH1 (Figure 1A). These results suggested that GBZ skews TLR4 activation by reducing the intensity of the IRF3 signaling pathway (22), while promoting instead the MyD88-dependent pathway leading to increased transcription of IL-12 and potentially also of IL-10 (23). When cytokines expression in response to *Proteus* was monitored, we could observe a modest reduction in IL-6 production, while IL-12 p70 and IL-10 expression was strongly augmented both at the transcription and secretion levels (Figures 1C,D), confirming the microarray analysis results. When cytokines secretion was evaluated after poly I:C stimulation instead of *P. mirabilis* (Figure 1D), an inhibitory effect of GBZ on TLR3 activation, which depends solely on TRIF for its signal transduction (24, 25), was observed, confirming that the drug likely interferes with the TRIF-dependent pathway and could have previously unexploited anti-inflammatory activity.

### GBZ Prevents d-galN/LPS-Induced Lethality

**Figure 2:**
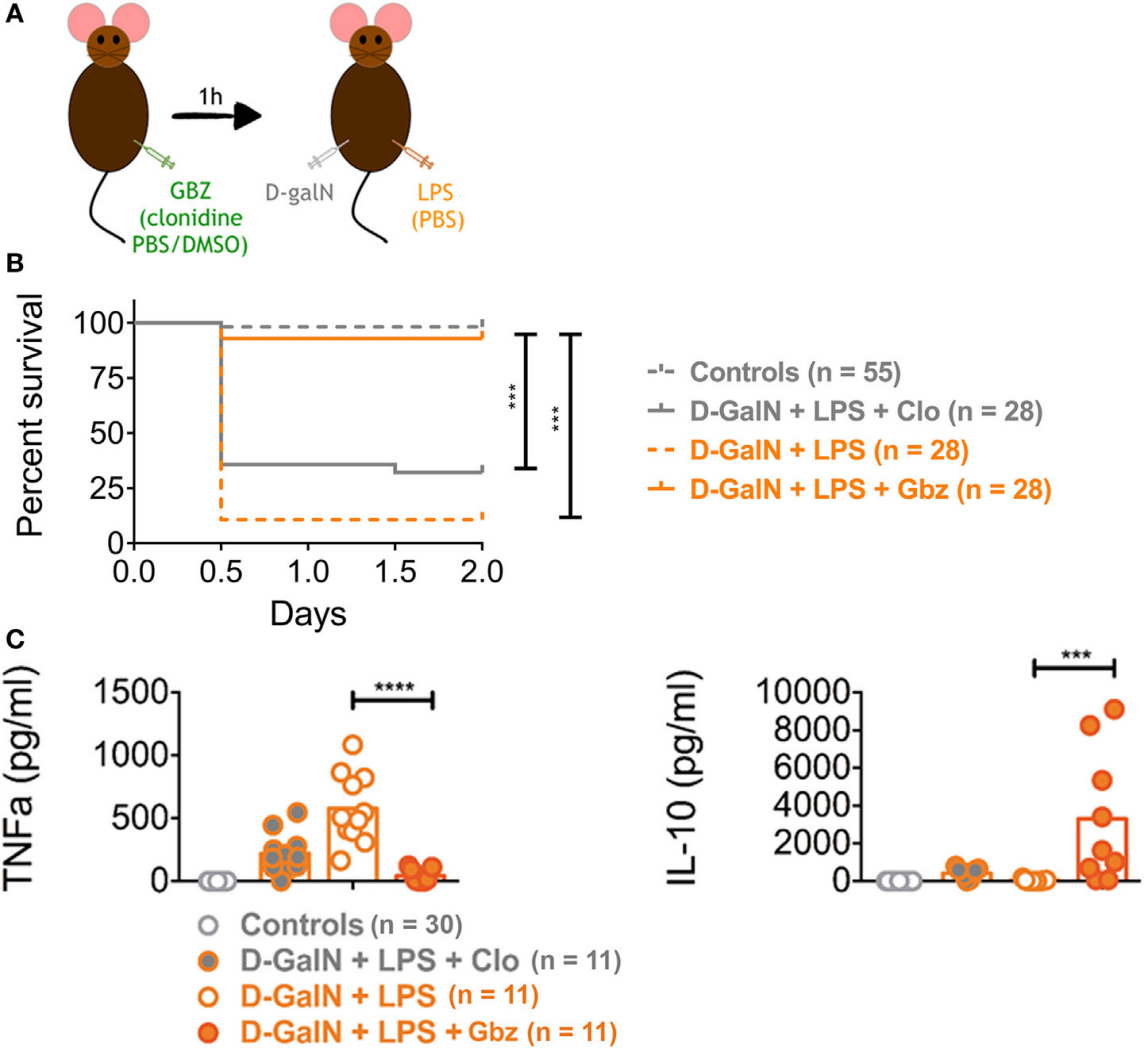
Guanabenz (GBZ) prevents lipopolysaccharide (LPS)-dependent lethality. C57BL/6 wild-type mice were injected with GBZ or clonidine (2 mg/kg of mouse), followed by d-galactosamine (d-galN) (20 mg/mouse) and LPS or PBS for control mice (20 ng/20 g of mouse) **(A)**. The mice survival is represented in a Kaplan–Meier plot. Statistical analysis on survival curves were performed using log-rank test, followed by Benjamini–Yekutieli correction (***p < 0.001) **(B)**. The concentration of circulating tumor necrosis factor-α (TNF-α) and interleukin-10 (IL-10) was measured 1 and 3 h after LPS injection, respectively. Statistical significance was assigned using one-way ANOVA test followed by Tukey range test to assess the significance among pairs of conditions (***p < 0.001, ****p < 0.0001) **(C)**.

These *in vitro* results, indicating that IL-10 production can be enhanced by GBZ, confirmed that our strategy of targeting eIF2α phosphorylation to limit systemic inflammation and liver failure during endotoxemia was appropriate. We, therefore, investigated further GBZ activity *in vivo*, using the classical d-galN-dependent lethality model induced by LPS. Mice were injected with GBZ i.p. (2 mg/kg), 1 h before administration of *E. coli* O55:B5 LPS (20 ng) (Figure 2A). The potential side effects of the α2-adrenergic receptor agonist activity of GBZ were controlled in a parallel cohort by injecting clonidine instead of GBZ. Clonidine [*N*-(2,6-dichlorophényl)-4,5-dihydro-1*H*-imidazol-2-amine] belongs to a different class of chemical compounds with an affinity similar to GBZ for the α2-adrenergic receptor, but without any eIF2-α phosphorylation enhancing activity (26). In this experimental context, a mortality of 75–95% was observed after 2 days in the LPS-injected C57BL6 mice cohorts, co-injected or not with clonidine, thus demonstrating the endotoxin lethality and ruling out a strong protective effect of induced hypotension in this model. GBZ administration had however a stunning impact and prevented this negative outcome by restoring 98% of mice viability after LPS injection (Figure 2B). Interestingly, at these doses of LPS, follow-up experiments over 5 days, never revealed any additional mortality than the one observed within 48 h of the injection, and we therefore reduced our experimental observation times to a maximum of 2 days postinjection.

Given the extremely high efficiency of protection provided by GBZ *in vivo*, we next monitored circulating TNF-α levels to evaluate whether GBZ administration impairs cytokines secretion in LPS-injected mice. Upon blood collection at several time points, circulating cytokines were quantified by ELISA (Figure 2C; Figure S1 in Supplementary Material). A significant decrease in the peak of TNF-α production was observed after 1 h of LPS injection, while the secretion of the anti-inflammatory cytokine IL-10 was strongly augmented after 3 h in mice treated with GBZ (Figure 2C). These data confirmed the results obtained with DCs *in vitro* and suggested that GBZ dampens LPS-induced pro-inflammatory reaction though both a reduction in TNF-α secretion and increased IL-10 production. We noticed that clonidine, even though not as effective as GBZ in rescuing viability, could mildly reduce TNF-α secretion, potentially by reducing the systemic distribution of LPS and cytokines, further suggesting that the hypotensive activity of GBZ could also contribute to endotoxemia reduction.

**Figure 3:**
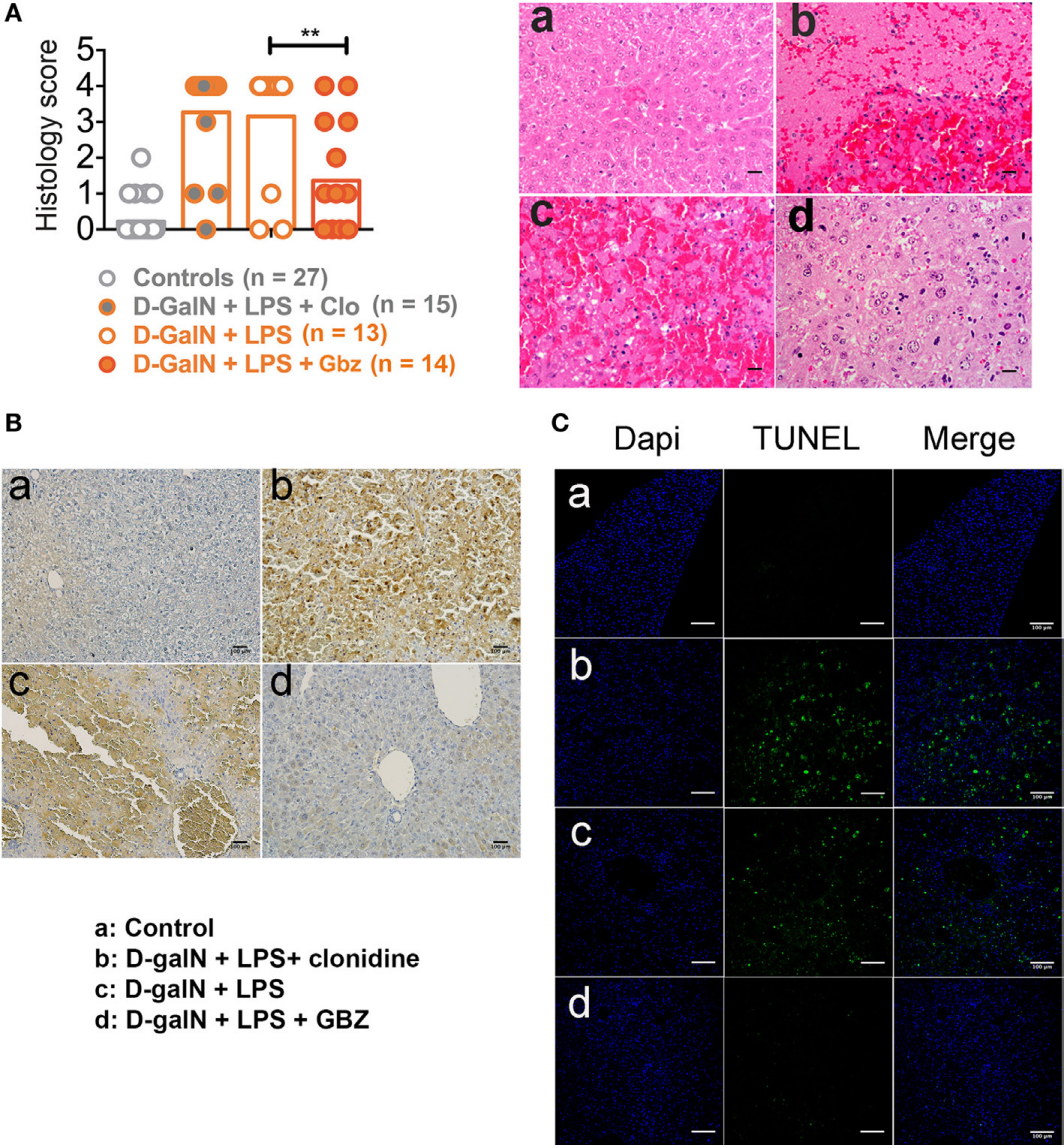
Guanabenz (GBZ) prevents lipopolysaccharide (LPS)-induced liver damage. **(A)** Livers were collected within few hours of death or after 48 h and the histological score for tissue damage was determined as indicated in Section “Materials and Methods.” Representative image for each condition is shown with a magnification of 20× (scale bar = 100 µM). a: Control; b: d-galactosamine (d-galN) + LPS + clonidine; c: d-galN + LPS; d: d-galN + LPS + GBZ. Statistical significance was assigned using one-way ANOVA test (**p < 0.01) **(A)**. Immunohistology staining for caspase 3 (brown) showing massive accumulation of the cleaved caspase upon endotoxemia, which was prevented by GBZ treatment (scale bar = 100 μM) **(B)**. TUNEL staining (green) reveals the accumulation of apoptotic cells (dapi in blue) upon endotoxemia, which was profoundly reduced by GBZ treatment (scale bar = 100 μM) **(C)**.

In the d-galN/LPS model, lethality is mostly caused by a caspase 3-dependent fulminant apoptotic hepatitis induced by TNF-α. We thus evaluated the protective effect of GBZ on liver by performing an anatomopathological analysis of mice 2 days after endotoxin injection or within few hours of their death. Histological analysis of liver section using hematoxylin-eosin staining and pathological scores (see Materials and Methods) indicated that GBZ treatment protected the liver from severe injuries induced by LPS (Figure 3A). When hepatocytes apoptosis was evaluated using activated caspase 3 immunostaining and terminal deoxynucleotidyl transferase dUTP Nick-End labeling (TUNEL) (Figures 3B,C), we could observe a massive accumulation of activated caspase 3 and of apoptotic cells in the liver section of LPS-injected mice, while the livers of GBZ/LPS treated animals remained only very lightly affected. These contrasted results suggest that in addition of its capacity to increase IL-10 production, GBZ protects efficiently hepatocytes from TNF-α-induced apoptosis.

### GBZ Prevents d-galN/LPS-Induced Lethality Independent of Hypotension

**Figure 4:**
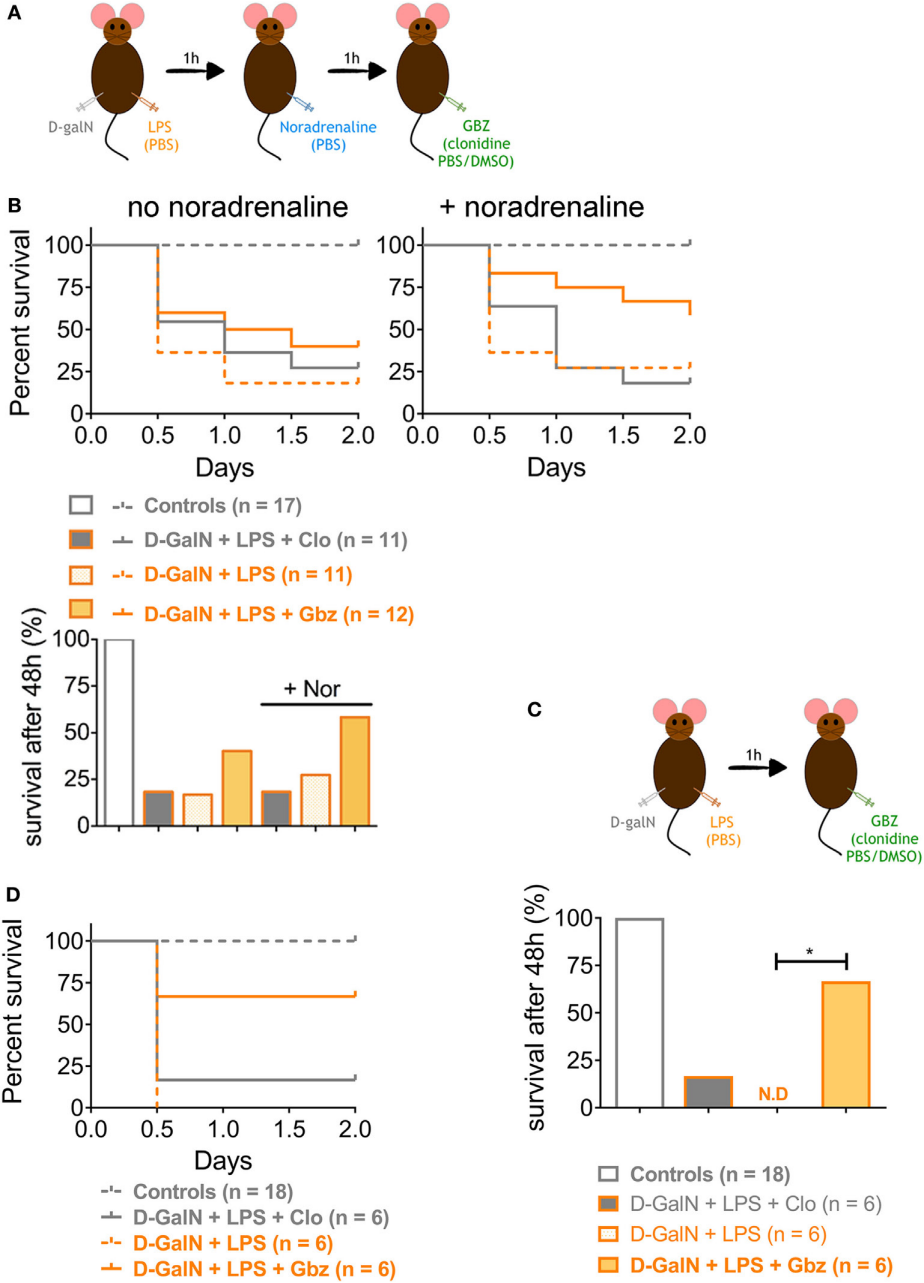
Guanabenz (GBZ) rescues mice from lipopolysaccharide (LPS)-induced lethality independently of blood hypotension. **(A,B)** C57BL/6 wild-type mice were injected with d-galactosamine (d-galN) (20 mg/mouse) and LPS or PBS for control mice (20 ng/20 g of mouse). After 1 h, mice were injected with noradrenaline or PBS for control mice (0.5 mg/kg). GBZ or clonidine (2 mg/kg of mouse) were injected 1 h after noradrenaline. **(B)** The mice survival is represented as total percentage of survival after 48 h, or in a Kaplan–Meier plot. Statistical analysis on survival curves were performed using log-rank test, followed by Benjamini–Yekutieli correction. **(C,D)** C57BL/6 wild-type mice were injected with d-galN (20 mg/mouse) and LPS or PBS for control mice (20 ng/20 g of mouse). After 1 h, mice were injected with GBZ or clonidine (2 mg/kg of mouse). **(D)** The mice survival is represented as total percentage of survival after 48 h, or in a Kaplan–Meier plot. Statistical analysis represented on the histogram, was performed using log-rank test on the survival curves, followed by Benjamini–Yekutieli correction (*p < 0.05).

**Figure 5:**
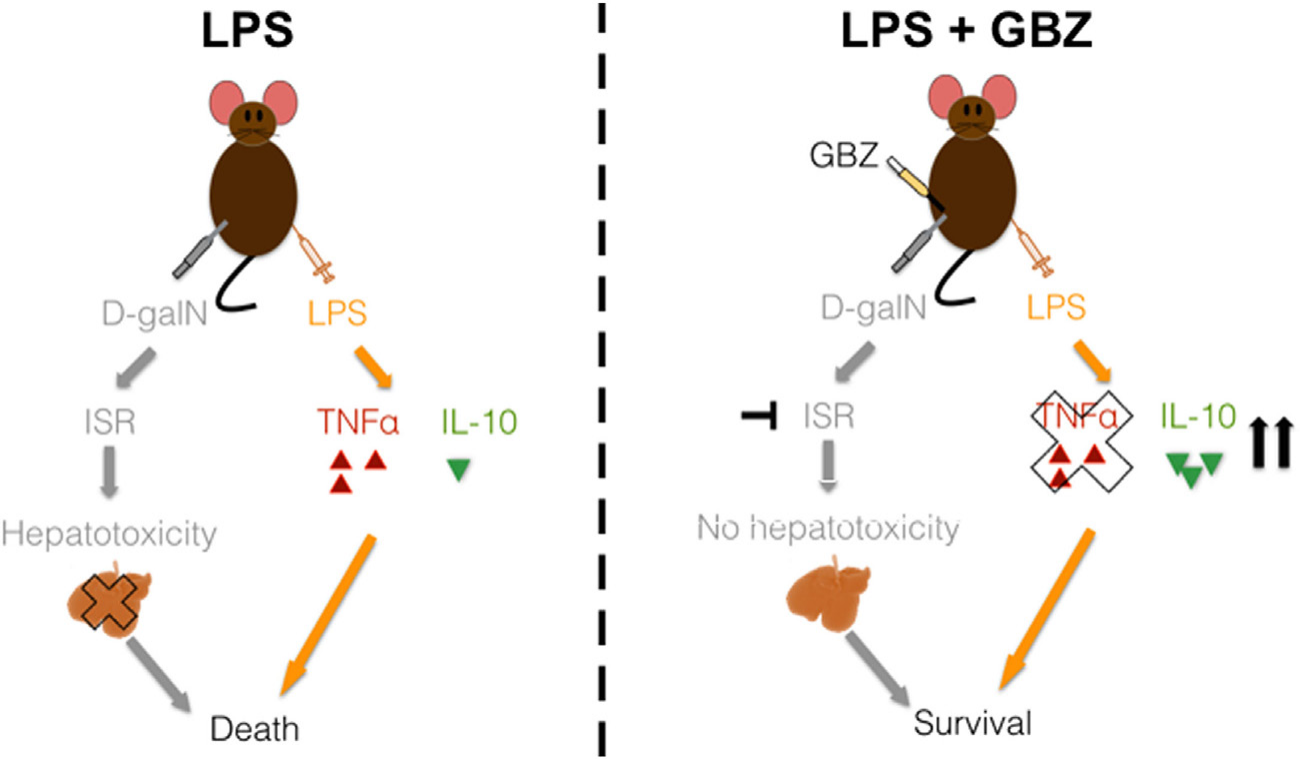
Model of Guanabenz (GBZ) effect in the lipopolysaccharide (LPS)/d-galactosamine (d-galN) model. Our results led to the model above: when injected with LPS and d-galN, mice experience an integrated stress response (ISR) that leads to massive apoptosis in the liver. At the same time, the detection of LPS creates an over-inflammation state. These two elements provoke the final death of the animal. When mice are treated with GBZ (at an interval of 1 h max after the septic shock), this drug is able to block both the ISR (thus the liver injury) and to switch the immune response from pro-inflammatory to anti-inflammatory, rescuing the animal’s survival.

We demonstrated that LPS-induced lethality can be prevented by administrating GBZ 1 h prior endotoxin injection. However, based on the results obtained with clonidine, the hypotensive activity of the compounds seems to contribute partially to mouse survival in the d-galN/LPS experimental model. To investigate this issue and evaluate further the GBZ curative potential post-LPS administration, the vasopressor l-noradrenaline was injected i.p. 1 h after LPS administration to compensate for the hypotension induced both by LPS and GBZ or clonidine delivery (Figure 4A). As expected, l-noradrenaline administration alone was not able to ameliorate mice survival (Figure 4B) confirming that LPS-induced hypotension is not a leading cause of death in this model (6). GBZ treatment 2 h post-LPS injection rescued partially mice from LPS-induced lethality (40%) (Figure 4B), however, with an efficiency less pronounced than when the inhibitor was delivered preventively. When l-noradrenaline was administrated 1 h after LPS, GBZ synergized with the vasopressor and significantly increased mouse resistance to LPS, reaching 60% of survival after 48 h (Figure 4B). Conversely, clonidine lost its mild effect on viability when administrated after noradrenaline, confirming that induced hypotension likely delays the distribution of injected endotoxins and cytokines, but has no major impact on liver protection nor induction of cytokine expression (Figure 4B). The lesser efficacy of GBZ delivery 2 h post-LPS injection could be explained by the kinetic of TNF-α production, strongly peaking 1 h after LPS injection (Figure 2D), and thus unlikely to be inhibited by a later administration of GBZ. When, GBZ was instead injected only 1 h after the endotoxin to match the kinetics of TNF-α production (Figure 4C), survival in the LPS/GBZ cohort increased to 67% (Figure 4D), demonstrating that GBZ can conserve its protective effect during ongoing endotoxemia. Taken together, these results indicate that GBZ could represent an interesting option to reduce LPS-induced liver damage, provided that blood pressure is controlled (Figure 5), as it is generally the case in emergency care conditions, during which noradrenaline-like vasopressors are injected to re-establish blood pressure levels in patients (27).

## Discussion

Multi-organ failure in response to uncontrolled microbial infection is characterized by low blood pressure accompanied by massive pro-inflammatory cytokines release and liver damage (28). The ISR has been recently shown to be the main cause of liver failure due to uncontrolled hepatocytes apoptosis triggered by TNF-α, as demonstrated for several liver diseases, such as acute liver injury, obesity-associated fatty liver, and viral hepatitis (8). GBZ, a clinically approved chemical compound to treat hypertension, has been shown to amplify eIF2α-phosphorylation during the ISR by interfering with the negative feed-back activity of GADD34. Consequently, GBZ slows down protein synthesis recovery during stress and prolongs the ISR in the crucial period during which damaged cells or tissues initiate apoptosis. In line with these observations, GBZ administration has been reported to be beneficial in different clinical conditions involving the ISR, such as amyotrophic lateral sclerosis (29) or multiple sclerosis (30). We thus evaluated the capacity of GBZ to alter both the capacity of immune cells to respond to endotoxin, as well as to prevent apoptosis in the liver of mice submitted to LPS/d-galN-induced lethality. Microarray analysis was used here to monitor globally the impact of GBZ on immune cells reacting to gram-negative bacteria or LPS and establish which fraction(s) of their function could be affected by this drug. The results of this microarray experiment confirmed that GBZ skewed the response of immune cells to LPS toward producing more IL-10, raising the possibility that in addition of impacting apoptotic cell survival, the drug could have strong anti-inflammatory activity that should be confirmed *in vitro* and explored further *in vivo*.

Our result *in vitro* obtained with DCs suggests that GBZ reduces TRIF-dependent signaling during TLR4 stimulation, thus augmenting NFκ-B-dependent transcription and promoting IL-10 production in LPS-stimulated cells. This observation was confirmed *in vivo*, during which a significant increase in IL-10 production was observed together with a strong impairment of circulating TNF-α levels upon GBZ pretreatment of LPS/d-galN-injected mice. GBZ immunomodulatory activity is, therefore, likely to be exerted through the combination of these two opposite events that skew the immune equilibrium toward an IL-10-dependent anti-inflammatory state (31). In our experimental model, survival correlates strongly with reduced liver damage, which could represent the main cause for the reduction in TNF-α levels observed upon GBZ treatment. GBZ prevents LPS-dependent liver destruction by reducing hepatocyte apoptosis; however, at this stage, we cannot distinguish between a protection from cell death exerted directly on hepatocytes by GBZ’s interference with the ISR, or a reduction in TNF-α levels production by stressed liver cells, that diminish considerably LPS-induced damages. Although a genetic deficiency in GADD34 increases hepatocytes apoptosis (32), reversible pharmacological inhibition by GBZ displays a protective phenotype, probably due to its rapid plasma clearance (half-life 1.8 h) and limited effect over eIF2α phosphorylation, preventing long-term GADD34 inactivation in the liver and associated proapoptotic CHOP expression (7). Our work also demonstrates that targeting eIF2-α phosphorylation represents a novel pharmacological option to interfere with endotoxemia both by reducing liver damage and altering TLR4-dependent cytokine release. Importantly, the hypotensive effect of GBZ might have some advantages in preventing the negative impact of cytokine during infection. However, the synergy observed with noradrenaline administration suggests that GBZ derivatives, lacking hypotensive activity, but capable of treating both proteotoxicity and associated inflammatory responses (33), could represent a new line of pharmacological agents for the critical care of patients suffering from bacterial-induced liver failure.

## Ethics Statement

This study was carried out in strict accordance with the recommendations in the Guide for the Care and Use of Laboratory Animals the French Ministry of Agriculture and of the European Union. Mice were housed under specific pathogen-free conditions and handled in accordance with French and European directives. Animals were housed in the CIML animal facilities accredited by the French Ministry of Agriculture to perform experiments on alive mice. All animal experiments were approved by Direction Départementale des Services Vétérinaires des Bouches du Rhône (Approval number A13-543). All efforts were made to minimize animal suffering. The experiments were performed with C57BL/6 mice purchased from Janvier. Sex and age were varying according to the experiment performed. Name of the project for the ethics committee: Impact du stress sur la synthèse protéique conséquence sur l’immunité et la mort cellulaire. Evaluated by: C2EA-14 Comité d’éthique en expérimentation animale de Marseille.

## Author Contributions

JP, EG, and PP designed research, analyzed data, and wrote the paper. JP, CB, VC, AM, and LC performed research. CL performed the anatomopathological analysis. JP and PP performed bioinformatics analysis. JP and LS performed statistical analysis.

## Conflict of Interest Statement

The authors declare that the research was conducted in the absence of any commercial or financial relationships that could be construed as a potential conflict of interest.

## References

[1] Rittirsch D Flierl MA Ward PA. Harmful molecular mechanisms in sepsis. Nat Rev Immunol (2008) 8:776–87. 10.1038/nri2402 18802444 PMC2786961

[2] Kagan JC Defining the subcellular sites of innate immune signal transduction. Trends Immunol (2012) 33:442–8. 10.1016/j.it.2012.06.005 22817912 PMC3427413

[3] Yan J Li S Li S The role of the liver in sepsis. Int Rev Immunol (2013) 33:498–510. 10.3109/08830185.2014.889129 PMC4160418 24611785

[4] Gustot T Durand F Lebrec D Vincent JL Moreau R Severe sepsis in cirrhosis. Hepatology (2009) 50:2022–33. 10.1002/hep.23264 19885876

[5] Silverstein R. d-galactosamine lethality model: scope and limitations. J Endotoxin Res (2004) 10(3):147–62. 10.1179/096805104225004879 15198850

[6] Mignon A Rouquet N Fabre M Martin S Pagès JC Dhainaut JF LPS challenge in d-galactosamine-sensitized mice accounts for caspase-dependent fulminant hepatitis, not for septic shock. Am J Respir Crit Care Med (1999) 159:1308–15. 10.1164/ajrccm.159.4.9712012 10194182

[7] Rao J Zhang C Wang P Lu L Qian X Qin J C/EBP homologous protein (CHOP) contributes to hepatocyte death via the promotion of ERO1α signalling in acute liver failure. Biochem J (2015) 466:369–78. 10.1042/BJ20140412 25387528

[8] Malhi H Kaufman RJ Endoplasmic reticulum stress in liver disease. J Hepatol (2011) 54:795–809. 10.1016/j.jhep.2010.11.005 21145844 PMC3375108

[9] Chen N Nishio N Ito S Tanaka Y Sun Y Isobe KI. Growth arrest and DNA damage-inducible protein (GADD34) enhanced liver inflammation and tumorigenesis in a diethylnitrosamine (DEN)-treated murine model. Cancer Immunol Immunother (2015) 64:777–89. 10.1007/s00262-015-1690-8 25832002 PMC11029570

[10] Inaba Y Furutani T Kimura K Watanabe H Haga S Kido Y Growth arrest and DNA damage-inducible 34 regulates liver regeneration in hepatic steatosis in mice. Hepatology (2015) 61:1343–56. 10.1002/hep.27619 25420998

[11] Clavarino G Cláudio N Couderc T Dalet A Judith D Camosseto V Induction of GADD34 is necessary for dsRNA-dependent interferon-β production and participates in the control of chikungunya virus infection. PLoS Pathog (2012) 8(5):e1002708 10.1371/journal.ppat.1002708 22615568 PMC3355096

[12] Clavarino G Cláudio N Dalet A Terawaki S Couderc T Chasson L Protein phosphatase 1 subunit Ppp1r15a/GADD34 regulates cytokine production in polyinosinic:polycytidylic acid-stimulated dendritic cells. Proc Natl Acad Sci U S A (2012) 109:3006–11. 10.1073/pnas.1104491109 22315398 PMC3286954

[13] Dalet A Argüello RJ Combes A Spinelli L Jaeger S Fallet M Protein synthesis inhibition and GADD34 control IFN-β heterogeneous expression in response to dsRNA. EMBO J (2017) 36(6):761–82. 10.15252/embj.201695000 28100675 PMC5350567

[14] Tsaytler P Harding HP Ron D Bertolotti A. Selective inhibition of a regulatory subunit of protein phosphatase 1 restores proteostasis. Science (2011) 332:91–4. 10.1126/science.1201396 21385720

[15] Neuber C Uebeler J Schulze T Sotoud H El-Armouche A Eschenhagen T. Guanabenz interferes with ER stress and exerts protective effects in cardiac myocytes. PLoS One (2014) 9(6):e98893. 10.1371/journal.pone.0098893 24892553 PMC4044035

[16] Ohri SS Mullins A Hetman M Whittemore SR Inhibition of GADD34, the stress-inducible regulatory subunit of the endoplasmic reticulum stress response, does not enhance functional recovery after spinal cord injury. PLoS One (2014) 9(11):e109703 10.1371/journal.pone.0109703 25386686 PMC4227638

[17] Way SW Podojil JR Clayton BL Zaremba A Collins TL Kunjamma RB Pharmaceutical integrated stress response enhancement protects oligodendrocytes and provides a potential multiple sclerosis therapeutic. Nat Commun (2015) 6:6532. 10.1038/ncomms7532 25766071 PMC4360920

[18] Holmes B Brogden RN Heel RC Speight TM Avery GS. Guanabenz: a review of its pharmacodynamic properties and therapeutic efficacy in hypertension. Drugs (1983) 26:212–29. 10.2165/00003495-198326030-00003 6352237

[19] Siegmund B Lear-Kaul KC Faggioni R Fantuzzi G Leptin deficiency, not obesity, protects mice from Con A-induced hepatitis. Eur J Immunol (2002) 32:552–60. 11828372 10.1002/1521-4141(200202)32:2<552::AID-IMMU552>3.0.CO;2-H

[20] Cláudio N Dalet A Gatti E Pierre P. Mapping the crossroads of immune activation and cellular stress response pathways. EMBO J (2013) 32:1214–24. 10.1038/emboj.2013.80 23584529 PMC3642686

[21] Lee MN Ye C Villani AC Raj T Li W Eisenhaure TM Common genetic variants modulate pathogen-sensing responses in human dendritic cells. Science (2014) 343:1246980. 10.1126/science.1246980 24604203 PMC4124741

[22] Negishi H Yanai H Nakajima A Koshiba R Atarashi K Matsuda A Cross-interference of RLR and TLR signaling pathways modulates antibacterial T cell responses. Nat Immunol (2012) 13:659–66. 10.1038/ni.2307 22610141

[23] Rutz S Ouyang W. Regulation of interleukin-10 expression. Adv Exp Med Biol (2016) 941:89–116. 10.1007/978-94-024-0921-5_5 27734410

[24] Akira S Akira S Uematsu S Uematsu S Takeuchi O Takeuchi O Pathogen recognition and innate immunity. Cell (2006) 124:783–801. 10.1016/j.cell.2006.02.015 16497588

[25] Kagan JC Signaling organelles of the innate immune system. Cell (2012) 151:1168–78. 10.1016/j.cell.2012.11.011 23217704 PMC3523748

[26] Ruffolo RR Jr. Nichols AJ Stadel JM Hieble PJ Structure and function of a-adrenoceptors. Pharmacol Rev (1991) 43:475. 1685567

[27] Avni T Lador A Lev S Leibovici L Paul M Grossman A. Vasopressors for the treatment of septic shock: systematic review and meta-analysis. PLoS One (2015) 10(8):e0129305. 10.1371/journal.pone.0129305 26237037 PMC4523170

[28] Angus DC van der Poll T Severe sepsis and septic shock. N Engl J Med (2013) 369:840–51. 10.1056/NEJMra1208623 23984731

[29] Wang L Popko B Tixier E Roos RP. Guanabenz, which enhances the unfolded protein response, ameliorates mutant SOD1-induced amyotrophic lateral sclerosis. Neurobiol Dis (2014) 71:317–24. 10.1016/j.nbd.2014.08.010 25134731 PMC4179984

[30] Way SW Popko B Popko B Way SW Harnessing the integrated stress response for the treatment of multiple sclerosis. Lancet Neurol (2016) 15:434–43. 10.1016/S1474-4422(15)00381-6 26873788 PMC4792730

[31] Wiersinga WJ Leopold SJ Cranendonk DR van der Poll T. Host innate immune responses to sepsis. Virulence (2014) 5:36–44. 10.4161/viru.25436 23774844 PMC3916381

[32] Ito S Tanaka Y Oshino R Okado S Hori M Isobe KI. GADD34 suppresses lipopolysaccharide-induced sepsis and tissue injury through the regulation of macrophage activation. Cell Death Dis (2016) 7:e2219. 10.1038/cddis.2016.116 27171261 PMC4917654

[33] Das I Krzyzosiak A Schneider K Wrabetz L Antonio MD Barry N Preventing proteostasis diseases by selective inhibition of a phosphatase regulatory subunit. Science (2015) 348:229–32. 10.1126/science.aaa0193 25859045 PMC4490275

