# Supplementary figures and images for: Guanabenz Prevents d-Galactosamine/Lipopolysaccharide-Induced Liver Damage and Mortality
Source: Front Immunol. 2017 Jun 13;8:679. doi: 10.3389/fimmu.2017.00679 (PMC5468566; doi:10.3389/fimmu.2017.00679)

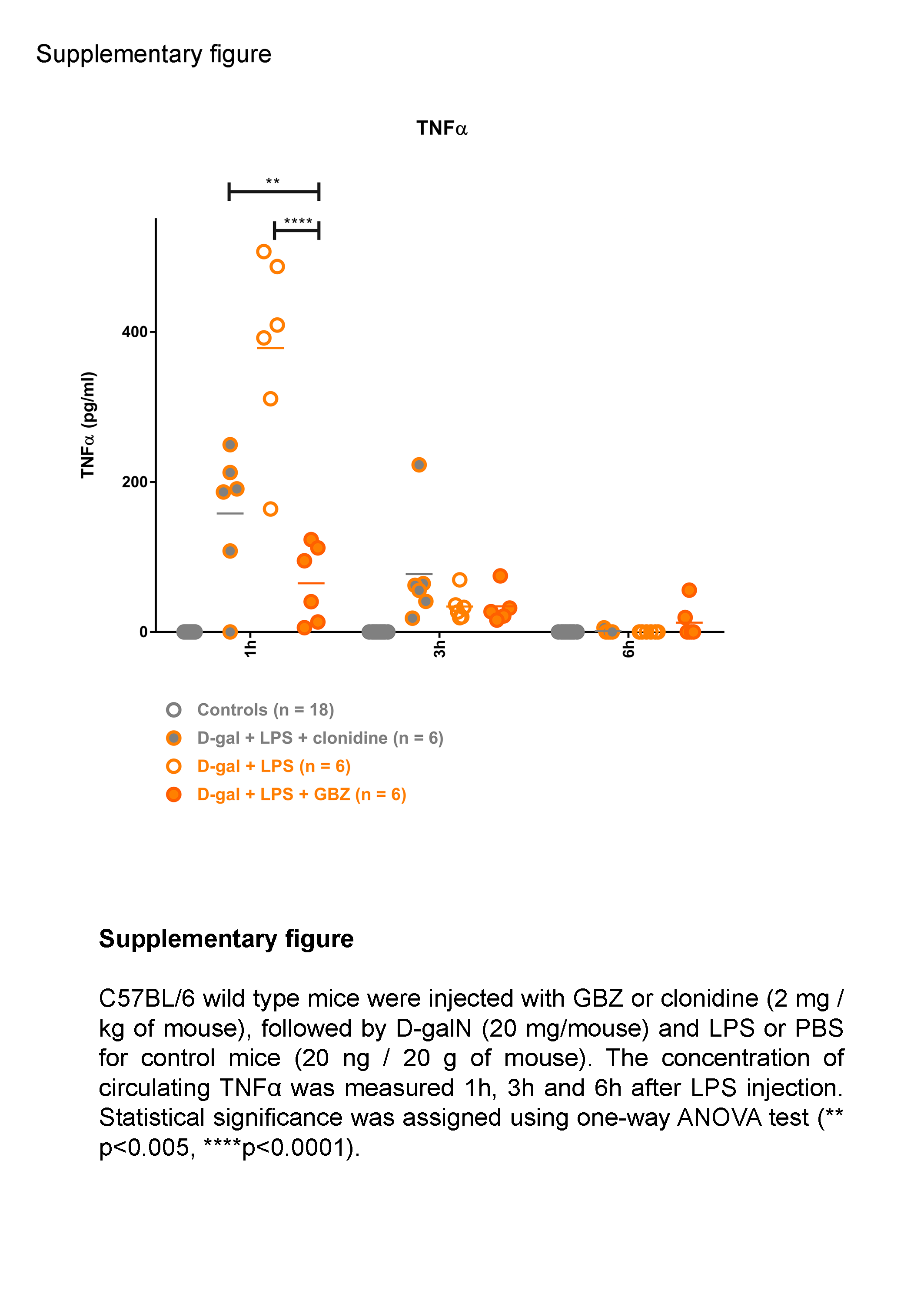

Supplement: Supplementary file 1 [file Image_1.TIF]
